# Developing a theory of change to improve the implementation of communication support for adults with severe learning disabilities in social care settings

**DOI:** 10.1186/s12913-026-15154-1

**Published:** 2026-08-06

**Authors:** Elizabeth Tuudah, Jill Bradshaw, Inez Gaczkowska, Victoria Ratti, Vicky Slonims, Kathy Leadbitter, Annette Bauer, Chris Hatton, Martin Knapp, Magdalena Mikulak, Alan Williams, Mary Busk, Perveen Quazi, Rosa A. Hoekstra

**Affiliations:** 1 Institute of Psychiatry, Psychology & Neuroscience, Department of Biostatistics & Health Informatics, King’s College London, London, SE5 8AB UK; 2 Intellectual Disabilities Research Institute, School of Social Policy and Society University of Birmingham, Edgbaston, Birmingham, B15 2TT UK; 3 Institute of Psychiatry, Psychology & Neuroscience, Department of Child and Adolescent Psychiatry, King’s College London, Evelina London Children’s Hospital, 1 Lambeth Palace Road, London, SE1 7EU UK; 4 University of Manchester, Oxford Rd, Manchester, M13 9PL UK; 5 London School of Economics and Political Science, PAN 8.01B, Pankhurst House, Houghton Street, London, WC2A 2AE UK; 6 Manchester Metropolitan University, Brooks Building, 53 Bonsall Street, Manchester, M15 6GX UK; 7 Lancaster University, Health Innovation Campus, Bailrigg, Lancaster, LA1 4YW UK; 8 Family Carers, London, UK; 9 Institute of Psychiatry, Psychology and Neuroscience, Department of Psychology, Guy’s Campus, King’s College London, London, SE1 1UL UK

**Keywords:** Learning disabilities, Nonverbal communication, Communication barriers, Implementation science, Program evaluation

## Abstract

**Background:**

Communication partners are crucial for adults with severe learning disabilities, as they recognise, interpret, and respond to verbal and non-verbal cues while sharing responsibility for creating a meaningful two-way communication process. Evidence-based strategies such as augmentative and alternative communication (AAC) can enhance interactions; however, their implementation in social care settings is inconsistent, constrained by workforce, organisational, and systemic barriers. This study–which forms part of the Improving Communication with Adults with Learning Disabilities (ICALD) programme–aimed to identify stakeholder-perceived barriers and facilitators to good communication support and to co-develop a Theory of Change (ToC) to guide implementation improvements in social care settings.

**Methods:**

Forty-eight stakeholders from diverse backgrounds, including adults with learning disabilities, participated in a total of 15 ToC workshops, one focus group, two interviews, and four supported conversations. Template Analysis based on the Consolidated Framework for Implementation Research (CFIR) was used to identify core themes, barriers, and facilitators across CFIR domains. These key findings informed the iterative, collaborative development of the ToC map.

**Results:**

Implementation barriers and facilitators identified across the CFIR domains indicated individual-, organisational-, and system-level factors influencing communication support. Key barriers included limited access to specialist professionals, inconsistent training, fragmented information-sharing systems, and variable staff knowledge of communication needs. Facilitators included strong leadership commitment, collaborative working with speech and language therapists and family carers, and peer mentoring. Supported conversations highlighted the importance of staff responsiveness to non-verbal cues. The resulting ToC map outlines short-, intermediate-, and long-term outcomes through three core pathways: (1) collaborative development of resources and training, (2) improved information sharing and recording, and (3) ongoing skills development, coaching, and supervision.

**Conclusions:**

Improving the implementation of good communication support for adults with severe learning disabilities is likely to depend on organisational commitment, leadership engagement, and structured workforce development supported by specialist input. The ToC developed provides a theoretically grounded framework for translating known facilitators into practical, context-specific strategies. It highlights the necessity of capacity-building within teams and organisational alignment with the communication rights of adults with severe learning disabilities to achieve sustainable, consistent and effective practice change.

**Supplementary Information:**

The online version contains supplementary material available at https://doi.org/10.1186/s12913-026-15154-1.

## Background

Adults with learning disabilities often experience communication difficulties. This can include difficulties with comprehension of communication [1].

The American Speech-Language-Hearing Association defines communication as:

the active process of exchanging information and ideas. Communication involves both understanding and expression. Forms of expression may include personalized movements, gestures, objects, vocalizations, verbalizations, signs, pictures, symbols, printed words, and output from augmentative and alternative (AAC) devices [2].

Many individuals with severe to profound learning disabilities have difficulties in using formal linguistic codes in any form and have inconsistent ways of communicating which means they are highly reliant on interpretations made by other people [3]. People with severe learning disabilities typically have high support needs, and often additional motor and sensory impairments [4]. In a review by Snell et al. [5] of intervention studies for children and adults with a severe intellectual disability, 66.4% of participants as identified as pre-linguistic (so before the individual is able to use formal, symbolic language), 51.7% as having emergent communication and a minority of 6% communicating using multiple spontaneous words.

Hearing impairments are common, with rates varying from 24 to 93%, with prevalence increasing with severity of learning disability [6]. In addition to complexities arising from difficulties with expression and comprehension, almost all (98.7%) of adults with learning disabilities have more than one physical health condition, the most common being visual impairment, obesity, epilepsy, constipation and gait disorders [7]. Impact on ability to communicate is likely to be multiplicative [8], including ability to recognise when someone is communicating and ability to understand environmental cues.

As a fundamental human right, communication provides one of the means through which people connect, express themselves, and participate in society [9]. The finding that having communication support needs is associated with a lower quality of life should mean that services prioritise communication supports [1]. However, we know from previous research [10] that support for communication is typically poorer for those with the greatest communication needs. Limited access to good communication support is one factor that leads to adults with severe and profound learning disabilities being especially vulnerable to social isolation and abuse [11–14]. They may be particularly reliant on communication partners in order to be socially included. Due to their communication challenges, they are often unable to communicate when something has gone wrong [15–19].

Communication partnerships consist of at least two people who “exchange ideas and interpret meanings” [20]. Communication partnerships also exist between people with severe to profound learning disabilities and those who care for them. People with severe and profound intellectual disabilities rely on subtle idiosyncratic, and often non-verbal behaviours to express themselves [21]. Subsequently, they are reliant on communication partners who play a crucial role in recognising, interpreting, and responding to the potentially communicative behaviours of people with severe and profound learning disabilities, often taking increased responsibility for creating a meaningful two-way process of communication [21].

Evidence-based strategies such as augmentative and alternative communication (AAC) including the use of visual supports and objects of reference, signing systems and other communication aids, can support communication [22, 23].

The American Speech-Language-Hearing Association define AAC as:

…all of the ways that someone communicates besides talking. Augmentative means to add to someone’s speech. Alternative means to be used instead of speech [24].

Aided forms of communication include those where the individual is reliant on something external e.g. pictures whereas unaided forms of communication are where the individual uses parts of their body (e.g. speech or manual signs) [25].

In their review of speech and language therapy for adults, Wood and Standen [26] concluded that there was insufficient evidence around effectiveness of speech and language therapy interventions due to the low quality of studies, but they did find some evidence of improvements. These interventions aimed to increase engagement and social interaction (e.g. Intensive Interaction), improve articulation (e.g. speech training), and/or aspects of AAC (e.g. PECS – Picture Exchange Communication System). Danker et al. [27] found that PECS was most commonly used for people with severe to profound intellectual disabilities to support functional communication, noting a range of barriers to their use, including others’ attitudes, behaviours and knowledge. This is important when delivering indirect interventions to communication partners.

Communication interventions with people with learning disabilities typically involves staff training [28]. A recent systematic review focused on people with profound intellectual and multiple disabilities [29] and found that staff training can improve staff communication and responsiveness, but few good quality interventions were found.

The effectiveness of communication interventions depends on consistent and skilled implementation by staff and carers. This requires commitment, time, prioritisation of communication needs - fitting this in alongside other elements of support (e.g. support around person care) [30], and support from speech and language therapists (SLTs) [31]. Yet, systemic barriers, including high staff turnover, inconsistent content, quality and quantity of training, and limited access to specialist professionals and resources, limit sustained implementation in residential social care settings [32, 33].

A realist review around good communication with adults with learning disabilities in long-term care settings [18] was conducted as part of the Improving Communication with Adults with Learning Disabilities study (ICALD; NIHR204416). This informed the development of an initial programme theory, which explains how communication support could be optimally designed and implemented for adults with learning disabilities in residential care. This theory is built around ten context–mechanism–outcome configuration statements that describe what works, for whom, and why. Together, they show how particular contextual features activate specific responses that lead to particular outcomes. They illustrate the dynamic interplay between staff attitudes, organisational conditions, and communication practices, and reveal how these mechanisms either support or hinder the effective implementation of communication support. Good communication support enables adults with learning disabilities to exchange information, intentions and emotions with communication partners in ways that support people to be involved in their daily lives. It is respectful and helps to build mutually satisfying relationships [14–17].

Findings from the realist review identified that staff beliefs about an individual’s capacity to communicate can influence the quality of interactions [34–40]. If communication attempts made by an individual with learning disabilities are overlooked or dismissed, opportunities for interaction are lost. Conversely, staff who believe that the individual has communication abilities are more willing to adapt their communication style, helping to foster trusting relationships. Additionally, organisational commitment and policies that create the conditions for staff to recognise, interpret, and respond to the communicative capacities of people with learning disabilities can lead to good support communication [39–41]. The realist review served to synthesise the theoretical knowledge (that gained from published studies) necessary to inform the framework of this study. However, translating this knowledge into a practical implementable framework requires stakeholder collaboration to refine and build upon these findings, informed by an Implementation Science perspective.

Theory of Change (ToC) is a participatory approach for developing complex interventions which provides a structured method of mapping causal pathways, linking activities to outcomes, and identifying contextual factors, mechanisms, and underlying assumptions [42, 43]. ToC is particularly valuable for developing interventions in social care settings, where contextual, organisational, individual, and relational factors shape implementation outcomes such as feasibility, sustainability, and acceptability [42]. The ToC development process typically involves a series of discussions with key stakeholders, including individuals with lived experience, carers, professionals, and people in positions of leadership. Through this collaborative and iterative process, long-term goals are defined, intermediate outcomes needed to achieve long-term goals are identified, and assumptions and activities are specified, providing a framework to guide implementation and evaluation.

### Aim

Using the ToC approach, this study aimed to:

- understand the barriers and facilitators perceived by stakeholders in providing good communication support to adults with learning disabilities in residential social care, and;
- identify the causal pathways and underlying mechanisms through which good communication support for adults with severe learning disabilities can be achieved, illustrating how specific intervention components and stakeholder-identified features connect in a causal chain to ensure sustained implementation.

## Methods

### Design

This study adopted a qualitative design using the ToC approach. We conducted a series of ToC workshops, semi-structured interviews, a focus group, and supported conversations to explore participants’ experiences and their views on how to improve the implementation of good communication support.

Workshops enabled collaborative development of the ToC, while interviews and the focus group provided in-depth and complementary perspectives on implementation experiences of communication support for adults with learning disabilities. Supported conversations were used to ensure the inclusion of adults with learning disabilities with complex communication needs.

### Participants

Table 1 details the participant sampling strategy, recruitment routes, and participant involvement across workshops and supported conversations. The inclusion and exclusion criteria for workshops and supported conversations are shown in Table 2. Reasons for ineligibility and/or exclusion of participants were documented. The organisation, sector and professional roles of staff participants are reported in Additional File 2, Table 1S. The demographic characteristics of participants are reported in Table 3. Forty-eight participants took part in the study, ranging in age from 18 to 85 years. All participants with learning disabilities in this sample used speech to participate in discussions; detailed communication profiles (e.g., use of AAC systems) were not systematically collected.

**Table 1:** Participant sampling strategy, recruitment routes, and involvement in workshops, supported conversations, interviews and focus groups

Process	Description
Sampling strategy	*Family carers of people with learning disabilities and professionals*: Purposive, convenience, and snowball sampling was used to recruit participants with relevant experience or expertise. *Adults with learning disabilities and family carers of people with learning disabilities*: Purposive sampling.
Recruitment routes	*Adults with learning disabilities*,* family carers of people with learning disabilities and professionals*: Via the research team’s contacts with organisations in England; organisational gatekeepers; professional interest groups; word of mouth; and an online expression-of-interest form or direct contact with the research team. *Professionals*: Directly contact via the team’s network; via word of mouth; or via professional special interest groups.
Workshops	Participants were invited to take part in at least one workshop and to attend subsequent workshops (up to four in total).
Individual supported conversations	Participants with complex communication difficulties were supported by a familiar carer to help them express their views in either workshops or a supported conversation.
Interviews	Participants who could not attend a workshop were invited to take part in at least one interview for each ToC workshop topic (up to four in total).
Focus groups	Family carers and professionals were invited to take part in a single focus group. This was open to both new participants and those who had previously attended ToC workshops or interviews.

**Table 2:** Inclusion and exclusion criteria for workshops and supported conversations

Inclusion criteria	Exclusion criteria
Adults with learning disabilities Workshops: - Aged eighteen and above. - Currently receiving support in social care settings in England. - Can communicate using speech or with the aid of augmentative communication systems that will allow them to contribute to the workshops.	Adults with learning disabilities Workshops and supported conversation: - Individuals with severe or profound communication difficulties that would prevent them from contributing to conventional workshops or taking part in an individual supported conversation.
Supported conversations: - Aged eighteen and above. - Currently receiving support in social care settings in England. - Complex communication difficulties - Unable to contribute to conventional group discussions.	
**Professionals/families**: - Aged eighteen and above. - Currently living/working in England. Currently supporting adults (directly or indirectly) with learning disabilities living in social care settings either in a professional or personal capacity.	

**Table 3:** Descriptive characteristics of participants

	*N*=48 (%)
**Stakeholder**	
Adults with learning disabilities Commissioner Direct support staff Family carer Professional Service manager Trainer	13 (27%) 1 (2%) 8 (17%) 6 (13%) 13 (27%) 5 (10%) 2 (4%)
**Race and Ethnicity**	
Asian/Asian British – Indian Black British Black or Black British – African Black or Black British – Caribbean Mixed – any other mixed background White – British White – Other Prefer not to say	1 (2%) 1 (2%) 6 (13%) 2 (4%) 1 (2%) 35 (73%) 1 (2%) 1 (2%)
Age*	
20-29 years 30-39 years 40-49 years 50-59 years 60 years and above	10 (21%) 14 (29%) 8 (17%) 8 (17%) 4 (8%)
**Gender**	
Female Male	35 (73%) 13 (27%)
**Region of England**	
East Midlands London North-East North-West South-East South-West Yorkshire & the Humber	1 (2%) 11 (23%) 5 (10%) 10 (21%) 1 (2%) 2 (4%) 18 (38%)

*Four participants declined to provide information on their age

### Data collection

Fifteen workshops, two interviews, one stakeholder focus group, and four supported conversations were conducted between October 2024 and May 2025. Group sizes ranged from two to seven participants (see Table 4). Sessions we held both in person and remotely and all were audio-recorded via Microsoft Teams with participants’ consent.

**Table 4:** ToC workshops, interviews, supported conversations and focus group attended by stakeholders

Mode of data collection	Stakeholder composition	Number of attendees	Facilitators
ToC workshop topic 1^1^	Adults with learning disabilities*	3 adults with learning disabilities	ET, IG
ToC workshop topic 1^1^	Mixed*	2 adults with learning disabilities, 1 professional, 1 service manager, 2 direct support staff	ET, IG
ToC workshop topic 1^1^	Family carers*	4 family carers	VS, VR
ToC workshop topic 1^1^ and 2^2^	Professionals	4 professionals	RH, VR
ToC workshop topic 1^1^ and 2^2^	Professionals	2 service managers	ET, IG
ToC workshop topic 1^1^ and 2^2^	Adults with learning disabilities*	3 adults with learning disabilities	ET, IG
ToC workshop topic 2^2^	Professionals	1 professional, 1 service manager	ET, IG
ToC workshop topic 2^2^	Direct support staff	4 support staff	ET, IG
ToC workshop topic 3^3^	Mixed	1 professional, 1 trainer	ET, IG
ToC workshop topic 3^3^	Mixed	2 professionals, 1 family carer	ET, IG
ToC workshop topic 3^3^	Mixed	1 family carer, 3 direct support staff, 1 service manager, 1 trainer, 1 professional	ET, VR
ToC workshop topic 3^3^	Professionals	1 team manager, 1 senior direct support worker	ET, IG
ToC workshop topic 4^4^	Family carer**	1 family carer	ET
ToC workshop topic 4^4^	Commissioner**	1 commissioner	ET
ToC workshop topic 4^4^	Mixed	1 family carer, 2 professionals, 1 team manager	ET, IG
ToC workshop topic 4^4^	Mixed	1 trainer, 2 professionals	ET, IG
ToC workshop topic 4^4^	Adults with learning disabilities*	4 adults with learning disabilities	ET, IG
Individual supported conversation	Adults with learning disabilities	4 adults with learning disabilities	ET, VR
Focus group	Family carers*	4 family carers	VS, VR

* Denotes workshops and the focus group that were conducted in-person rather than online

** Denotes interviews

^1^ Workshop topic 1: To explore desired changes in communication support, alongside identifying key

barriers and facilitators to implementation

^2^ Workshop topic 2: To identify workforce knowledge gaps, the conditions required for successful

implementation of communication support and to define the long-term goals of the intervention

^3^ Workshop topic 3: To map the key outcomes and actions needed over time to improve communication

support in social care settings

^4^ Workshop topic 4: To examine underlying assumptions and establish indicators of successful change

This study is grounded in Equality, Diversity, and Inclusion principles. Specifically, the core team of academic researchers and SLTs iteratively developed accessible materials used during the sessions—including presentation slides, discussion prompts, and follow-up resources—to ensure the format of all activities supported focused discussions on communication across diverse stakeholders. Materials were developed using plain English, Photosymbols, and tailored prompts to specifically accommodate participants with learning disabilities.

### ToC workshops

Participants, including adults with learning disabilities, family carers, and professionals were invited to participate in a series of collaborative workshops. Each workshop followed a semi-structured format, guided by a topic-specific framework (see Table 4), and typically progressed through three stages: [1] introduction to the topic and objectives; [2] facilitated group discussion and collaborative activities (e.g., brainstorming, mapping causal pathways); and [3] a feedback and summary phase to confirm key points.

Workshops were iterative, with subsequent sessions building on outputs from earlier workshops to progressively develop the ToC map. To support collaboration, participants contributed ideas using speech, which were recorded in real time by the co-facilitator using the Mural online collaboration platform [41], allowing visual mapping of themes and relationships between components. Summaries of emerging themes were presented at the end of each session for clarification and validation.

For online workshops, participants were sent a Microsoft Teams invitation link in advance, along with joining instructions and information about the session procedures, including how to access the meeting and arrangements for audio recording.

Taking into account power dynamics (i.e., differences in roles, authority, or perceived status that may influence participation), some workshops were restricted to a specific participant group while other workshops included a mix of participant types (see Table 4). Four out of fourteen workshops were conducted in person across three UK cities to maximise accessibility and align with participants’ geographical locations. In-person workshops were held in university premises and charity sites. These sessions took place in quiet, private rooms arranged to support comfort and engagement, with access to a projector to display the live mapping process. Each workshop, lasted up to two hours, and was facilitated by two researchers (E.T., I.G., R.A.H., V.R., V.S.), with one leading the discussion and the co-facilitator taking field notes throughout.

To support accessibility and engagement, easy-read prompt materials were provided to adults with learning disabilities, and facilitators used prompts and clarification techniques where needed.

### Supported conversations for participants with complex communication difficulties

A topic guide was used to facilitate individual conversations with adults with learning disabilities. The topic guide was adapted from the ToC workshop guide, with input from the research team (J.B., V.S., E.T., V.R.). Questions focused on key components of the ToC, with questions designed to explore participant’s perceived barriers and facilitators to good communication support, desired changes in the support they receive from staff, suggestions for how staff communication practices can be improved, and what outcomes they considered important (see Additional File 1). Participants were accompanied by a carer who knew the person with learning disabilities well. Their role was to support the participant to express their views. This included supporting the participant’s understanding by reframing questions to support engagement during the conversation.

All supported conversations were conducted in-person by the researchers (E.T., V.R.) across a range of settings (two at a day centre, one in a residential setting, and one on university premises), but always in a quiet, private rooms. The in-person format allowed the researchers to establish rapport and supported better interpret non-verbal communication (e.g. facial expression, gestures), which were key for accurately capturing the views of participants with complex communication needs. The researchers kept written field notes during and soon after each supported conversation. Contextual details and non-verbal cues (e.g., facial expressions, eye contact, body language) of the participant were documented to inform the data analysis. Insights from the supported conversations were incorporated into the development of the ToC by linking participants’ experiences to key ToC components.

### Interviews

Interviews and workshops were conducted as distinct data collection events. While the workshops employed collaborative group methods (e.g., visual mapping, group brainstorming focused on prioritisation), individual interviews served as a flexible alternative for stakeholders who were unable to attend a scheduled workshop. By mirroring the workshop topics (see Table 4), these interviews ensured that all participants could contribute insights regarding the implementation of communication support (see Additional File 1).

All semi-structured interviews were conducted online by E.T. Each interview began with an introduction to the topic and objectives, followed by discussion guided by an overview of the evolving ToC map to explore participants’ experiences and their recommendations for improving support for adults with learning disabilities. Interviews concluded with a feedback and summary phase to confirm key points.

### Focus group

In contrast to the iterative, collaborative, and mapping-based procedures used in the ToC workshops, the focus group followed a one-off, semi-structured discussion format aimed at generating in-depth experiential data. A stakeholder focus group was conducted with professionals and family carers to explore their experiences of communication interventions and outcome measures (see Additional File 1). These stakeholders were more likely to have direct experience of implementing communication interventions and using tools to measure communication outcomes in practice.

The session began with an introduction to the study aims, followed by facilitated discussion using open-ended questions to elicit detailed accounts of participants’ experiences, including perceived effectiveness, feasibility, and challenges associated with communication interventions and assessment tools. Unlike the ToC workshops, the focus group did not involve collaborative mapping or real-time visual synthesis of data but instead focused on generating in-depth narrative data through group interaction.

Participants were invited to attend an in-person session held in a UK city close to where most participants resided, at a charity venue and at a convenient time. The session took place in a quiet and private room arranged to support comfort and engagement, with access to a projector where required.

The focus group was facilitated by two researchers (V.S., V.R.), with one leading the discussion and the other taking detailed notes. The discussion was audio-recorded and later analysed thematically.

The focus group was conducted concurrently with the ToC workshops, supported conversations, and interviews while the initial ToC map was being developed. Data regarding participant experiences served a dual purpose: first, to inform workshop discussions on barriers and facilitators to implementation; and second, to refine the intervention components within the ToC map. By integrating these perspectives on the ease of using specific interventions and measures across different contexts, the researchers were able to ensure the final ToC map accurately reflected the requirements for effective communication support in practice.

### Process of refining components of the ToC map

After each workshop, the facilitators reviewed the minutes, draft ToC maps, and audio recordings to refine the map and its core components. The map was developed iteratively based on ToC workshops and meetings with the ICALD team, ensuring the map aligned with the ICALD programme’s scope, relevant literature, and key discussion themes. Required changes were made ahead of subsequent ToC workshops.

The process for refining the implementation ToC focused on achieving consensus on its components within the framework of the ICALD programme aims (See Additional File 2, Table 2S for ToC terminology and definitions). To ensure inclusive input, each workshop (after the first) began with a recap, inviting participants to reflect, disagree, or introduce new ideas based on discussions in previous workshops. Attendance variations across workshops were managed by summarising key themes and feedback in Mural, allowing subsequent versions of the ToC map to iteratively define the intervention’s causal pathways. Due to the technical nature of some ToC components (such as indicators) and varying stakeholder knowledge about workforce practices, it was not feasible to seek detailed input on every element from all groups (e.g., adults with learning difficulties). Findings from the workshops, interviews, supported conversations and the focus group were synthesised to refine the ToC map. This iterative development was further substantiated by insights from our realist review [19], which informed the development and the interpretation of the study findings.

### Data analysis

Template Analysis [44] was selected as the analytical method because it accommodates the use of a priori themes derived from established theoretical frameworks while retaining flexibility for inductive coding. This approach is particularly suited to implementation research, where theoretical frameworks provide systematic structure for identifying determinants of implementation, but context-specific factors may emerge that do not map directly onto predefined constructs. The Consolidated Framework for Implementation Research (CFIR) [45] informed the initial coding template, providing domains and constructs that support systematic assessment of contextual and organisational factors influencing implementation (see Table 5).

**Table 5:** Definition of CFIR domains

CFIR domain	Definition
1. Intervention Characteristics	The “thing” being implemented, e.g., a new staff training programme.
2. Outer Setting	The setting in which the Inner Setting exists, e.g., social care system.
3. Inner Setting	The setting in which the innovation is implemented, e.g., a residential care home.
4. Individuals	The roles and characteristics of individuals.
5. Implementation Process	The activities and strategies used to implement the innovation.

Adapted from Damschroder et al. [45].

Combining Template Analysis with the CFIR enabled the research team to map participants’ experiences onto established implementation constructs while remaining open to emergent themes; this provided a more nuanced understanding of implementation barriers and facilitators unique to communication support in social care settings.

E.T. led the analysis with collaboration from the wider research team. All workshop, focus group, interview and focus group activities were and transcribed verbatim by the researchers (I.G., V.R., E.T.). Data analysis was conducted alongside data collection following an iterative process. First, E.T. and I.G. familiarised themselves with the data by reading transcripts, revisiting facilitator notes, and listening to audio recordings. They then conducted line-by-line coding independently. 40% of transcripts were blind double-coded by E.T. and I.G.

The initial coding template was developed collaboratively from a sub-sample of five transcripts. E.T. and I.G. independently mapped codes to corresponding CFIR domains and constructs, then met to review codes, resolve discrepancies through discussion, and agreed on the initial template structure. Inductive coding was conducted in parallel to capture themes that did not map onto CFIR domains. The template was refined through iterative team discussions and transcript reviews. Input from R.A.H. and J.B. helped shape the ToC mapping process and ensured the coding template aligned with the CFIR, the study aims, and relevant communication support literature. This was achieved by drawing on their combined expertise in ToC development, qualitative methodology and SLT. Although inductive codes were generated throughout the analysis, the final template focused on themes directly related to communication support implementation; inductively identified themes that fell outside this scope were not retained.

Several strategies were employed to enhance rigour and mitigate potential biases. Blind double-coding of 40% of transcripts allowed discrepancies in interpretation to be identified and resolved through discussion. The iterative development of the coding template involved multiple researchers with different disciplinary perspectives, reducing the influence of individual assumptions. E.T. maintained a reflective diary throughout the analysis, documenting coding decisions and template modifications. Additionally, the research team regularly revisited audio recordings, transcripts, and field notes to verify interpretations and substantiate components of the ToC development. Input from team members not directly involved in data collection (e.g. J.B.) provided external perspective on the emerging analysis. Data analysis concluded when no new relevant themes were identified.

### Researcher positionality

The research team comprises researchers from UK higher education institutions (two of whom are also consultant SLTs), with expertise in communication support for people with learning disabilities, qualitative research methods, and co-developing iterative interventions with underrepresented groups using ToC and family carers. The professional and academic training of the team is rooted in Western perspectives on addressing communication challenges in social care. This shaped the development of the data collection materials, as well as the analysis and interpretation of findings. To mitigate potential biases arising from this positioning, the team incorporated regular reflexive discussions, maintained detailed audit trails of analytical decisions, and sought input from stakeholders throughout the ToC development process (see Additional File 2 for full details). No specific model of Speech and Language Therapy was drawn upon here, rather the focus emerged from discussions.

### Ethics

The study was approved by the West Midlands - Coventry & Warwickshire Research Ethics Committee (24/WM/0147; 31/07/2025), which is part of the Health Research Authority. This committee was selected because of their specialism in reviewing studies involving people who lack capacity. Informed consent (written or audio-recorded) was obtained before each participant’s first workshop or individual supported conversation. Consent was orally confirmed at each contact, and participants could opt-out from subsequent workshops at any time. If a participant lacked capacity to consent, researchers adhered to the Mental Capacity Act 2005 by obtaining written advice from a consultee to include them in the study, alongside the participant’s assent. All participants were debriefed following interviews, workshops, supported conversations and the focus groups in a conversation with the lead facilitator.

All identifying information was removed from transcripts to ensure anonymity. Each transcript was assigned a unique identification code and imported into NVivo (version 14) to facilitate coding and organisation of findings. All data, including field notes, were stored on a secure, restricted-access server, with access available to authorised research staff.

Adults with learning disabilities and family carers received NIHR recommended payments for their involvement in workshops and supported conversations [46] Professionals were offered a gift voucher for their participation.

## Results

### Barriers and facilitators to good communication support: stakeholders’ perspectives

Table 6 presents salient factors stakeholders perceived as barriers and facilitators to good communication support for adults with learning disabilities, mapped onto CFIR domains and described in detail below. The findings also informed the development of the ToC (see Additional File 3 for the full coding template).

**Table 6:** Overview of core themes and subcodes by CFIR domains

Domain	Theme	CFIR Construct	Subcode	Barrier	Facilitator
1. Innovation	1.1 Development and Testing	Innovation Source	Family carer input		x
			Communication champions	x	x
			Co-facilitate training with adults with learning disabilities		x
		Innovation Adaptability	Reviewing communication strategies	x	x
2. Outer Setting	2.1 Partnerships with external agencies	Partnerships & Connections	Referrals for specialist support	x	
	2.2 Availability of funding for communication training	Financing	Funding	x	
3. Inner Setting	3.1 Information Sharing	Access to Knowledge & Information	Knowledge sharing within the team	x	x
		Available Resources: Materials & Equipment	Access to appropriate resources	x	x
		Communications	Adults with learning disabilities’ assessments and documents	x	x
	3.2 Relationships	Relational connections	Importance of staff getting to know each adult with learning disabilities on a personal level	x	x
4. Individuals	4.1 Roles and responsibilities	Other Implementation Support	Organisational/senior management support	x	x
	4.2 Knowledge, skills and confidence	Capability	Staff knowledge and skills	x	x
			Understanding of non-verbal communication	x	x
5. Implementation Process	Integrating communication strategies	Doing	Trialling different strategies	x	x

### Domain 1: Innovation

#### Theme 1.1: Development and testing of communication interventions

This theme captures how communication support interventions are collaboratively refined through the involvement of experts and stakeholders to ensure interventions are both evidence-based and person centred.

#### Support from knowledgeable individuals: family carers, communication champions, SLTs

Social care staff emphasised the value of involving multiple stakeholders including family carers, SLTs, and increased staff resources to provide communication support. Employing “communication champions” (a term used by participants to describe dedicated roles that are not always formally reorganised as a standardised job title in the social care workforce) was one of the ideas offered to improve the understanding of individuals’ communication needs. Staff also considered access to guidance on interpreting communicative cues and gathering background information essential for allowing delivering person-centred support. Family carers were considered key sources of insight, particularly when staff were unfamiliar with the communication style of the people they supported.

If you don’t understand the resident very well, make sure you ask even the family… what is he trying to communicate?” (Direct support staff 04, ToC workshop)

Staff motivation and skillsets in practice leadership roles (staff responsible for guiding, supporting, and supervising staff to ensure high-quality, safe, and effective practice) were considered crucial, noting their influence on sharing knowledge and skills with the wider workforce:

… it depends on the [communication] champion… If somebody finds that that [modelling/teaching] is a difficult skill to have… it’s harder for them to then go in and have that right attitude. (Professional 09, ToC workshop)

#### Co-facilitate training with adults with learning disabilities

The involvement of adults with mild to moderate learning disabilities in co-facilitating staff communication training was identified as a key facilitator of effective support across all participant groups. Drawing on successful previous experience, some participants indicated this collaborative approach enhanced staff insight, empowered individuals with lived experience, and contributed to the development of more meaningful and impactful training.

…we do have our own people that we support that provide training alongside our trainers… it has been very successful… seeing and hearing… examples and difficulties from someone [with learning disabilities] is so, so valuable for our teams… it’s really empowering for the people that we support. (Manager 05, workshop)

#### Reviewing communication strategies

Staff observed that the effectiveness of strategies differed across individuals and often changed over time, highlighting the need for regular review and adaptation. Ongoing assessment and documentation of changes in communication needs were described as essential to ensuring responsiveness to individuals’ evolving needs. Flexibility in approach was emphasised to tailor strategies to individuals’ preferences and abilities.

It doesn’t have to fit into a box. Everybody’s different… [it’s] very easy to just do [the] same easy-read every time but not actually think about who is going to be reading it. (Professional 10, workshop)

### Domain 2: Outer domain

#### Theme 2.1: Partnerships with external agencies

This theme explores the impact of cross-sector collaboration, specifically the reliance on SLT expertise to provide clinical oversight and guidance related to communication support within social care settings.

#### Referrals for specialist support

Several professionals expressed concerns about limited access to SLTs, stating long waiting lists, sometimes spanning years. Support was often confined to brief input, leaving SLTs with few opportunities to evaluate the long-term effectiveness or ensure communication support strategies were fully embedded in practice. The lack of sustained involvement was perceived as a significant barrier to embedding and disseminating effective communication practices.

…the waiting lists are years long for communication… we’re lucky if we can offer three or four visits and then we have to say… ‘that’s all we can afford to give’ (Professional 01, workshop)

The provision of clear, external input, such as written reports from SLTs and their supervision were viewed by direct support staff as valuable tools for guiding implementation and enhancing practice within teams.

Giving us [a] kind of a report on their views as an outsider… it gives me the ability to go: “…this is what they’ve said. This is how we’re going to implement it, and I’ll show you how to do it”. (Direct support staff 10, workshop)

#### Theme 2.2: Availability of funding for communication training

This theme highlights how financial constraints and institutional funding priorities dictated the accessibility and long-term sustainability of upskilling the social care workforce in communication support skills.

### Funding

Funding constraints were seen as a major barrier to implementing and sustaining staff training in communication. Limited resources at both local authority and service levels were perceived to contribute to the prioritisation of mandatory care topics, such as safeguarding, over communication-specific training. Despite staff expressing a clear and ongoing need, communication training was often unavailable or deprioritised within existing social care training frameworks, compared with mandatory training such as safeguarding.

… I’ve been trying to get Makaton [a key-word signing system] for [our staff] the past nine years. And I still never been able to get… they can’t justify that expenditure. (Direct support staff 11, workshop)

Upskilling staff in roles to increase communication support and resources was also described as a pragmatic solution to limited SLT availability, with trained staff acting as a bridge between specialist expertise and frontline practice, helping to sustain effective communication support despite resource constraints.

*…the expectation [is] that we [SLTs] do more with less funding*,* or we do the same without the funding… I’m trying to break the cycle… by upskilling staff*,* … I train a core team that then filters down the information.* (Professional 09, workshop)

### Domain 3: Inner domain

#### Theme 3.1: Information sharing

This theme examines the internal systems and cultural practices that facilitate the exchange of knowledge regarding an individual’s communication needs across staff teams.

#### Knowledge sharing within the team

Knowledge related to supporting the communication needs of adults with learning disabilities was not consistently shared across staff teams, such as direct support staff and social care service managers. Knowledge transfer sometimes resembled a siloed approach which limited team-wide awareness and the implementation of effective communication practices.

…because I think a small proportion of staff get trained with, say, a certain client …that knowledge needs spreading across a wider staff team (Professional 12, workshop)

Participants considered collaborative knowledge sharing as essential for developing person-centred communication support. Through involving multiple staff and drawing on everyday interactions, a holistic understanding of individuals’ needs can be developed. Team-based reflection was seen to foster adaptive communication planning and support a culture of continuous and peer learning.

So being able to talk to all of the staff team about the same experiences, can then help develop that communication plan a lot better. (Direct support staff 10, workshop)

I think it’s also it’s good to hear other people’s opinions of the same thing… one person’s perspective of something that one person may think it’s working, that other person may think, it’s absolutely not working for me. (Direct support staff 11, workshop)

#### Access to appropriate resources

Staff reported access to centralised and trusted resource banks for social stories, symbol systems, and sign language videos as key for providing consistent communication support:

… a bank of where people can go to find resources… where people can use examples… social stories, videos of people doing basic sign language. (Trainer 02, workshop)

Ensuring that communication resources were physically accessible within care settings was also reported as crucial to supporting individuals’ day-to-day needs:

If he [the person I am supporting] is using symbols to communicate, I have to make sure that they are in his flat so that whenever he needs them, he must access them. (Direct support staff 07, workshop)

Embedding communication resources into daily practice through both digital infrastructure (i.e., organisational local networks used to share resources electronically) and physical accessibility equipped social care staff with consistently available tools that not only enhanced individualised support but also strengthened service-wide communication capacity in providing consistent support.

Several participants reported a lack of practical and accessible resources. Opportunities for meaningful interaction may be missed which highlights a broader organisational challenge in establishing coordinated systems that integrate relevant tools, expert guidance, and accessible information into routine practice.

There are things out there [communication resources], but you don’t know what you don’t know… it’s not at your fingertips… It’s hard to find… you can’t always get to really good resources. (Manager 03, workshop)

#### Adults with learning disabilities’ assessments and documents

Social care staff were expected by service managers and parents to engage proactively with communication documentation to ensure support aligned with the communication needs of adults with learning disabilities. While some participants reported consistently using these resources to inform their practice, others gave examples of insufficient review or comprehension of individuals’ communication support plans leading to communication challenges.

…sometimes I say “have you actually read everything on my son? I think you need to refresh”. (Family carer 03, workshop)

Participants perceived that the effectiveness of support was influenced by how well documentation captured individual communication preferences and was integrated into daily practice. Family members and professionals emphasised the importance of regularly consulting and refreshing knowledge from these documents to ensure responsive and person-centred communication.

In each resident’s house, they’ve got their file… how the resident communicates… we go through that file and read it thoroughly in order to understand more of the communication that my resident or my client normally uses on day-to-day basis. (Direct support staff 06, workshop)

#### Theme 3.2: Relationships

This theme focuses on the fundamental role of the relational bond between staff and adults with learning disabilities, which allows for the recognition of subtle, person-specific communication cues.

#### Importance of staff getting to know each adult with learning disabilities on a personal level

Several staff and family carers perceived understanding the individual preferences, cues, and subtle changes in emotional states of those they supported, through ongoing interactions, to effectively inform communication strategies. Observing and documenting nuances in communication and investing time to understand personal preferences outside of task-oriented interactions were seen as essential in building meaningful connections:

But it’s about the training of the individual staff… getting to know [them] on a one-to-one basis… over a period of time. What subtle changes mean. What’s happening now or why is he getting agitated or really happy. (Family carer 03, workshop)

Getting to know adults with learning disabilities on a personal level was described as essential for enhancing communication partnerships, as this relational knowledge enabled staff to recognise subtle cues and respond in ways that promoted connection and understanding.

### Domain 4: Individuals domain

#### Theme 4.1: Roles and responsibilities

This theme addresses the influence of leadership and management in establishing a culture that validates and prioritises communication support as a core staff responsibility.

#### Organisational/senior management support

Staff participants identified senior leadership as critical to embedding communication support within social care services. Organisational leads were seen as responsible for setting expectations, prioritising training, and cascading knowledge across teams.

…everyone needs to be involved in it [valuing the importance of communication support], but I think it needs to come from the top down as well (Trainer 02, workshop).

… knowledge of communication needs to, needs to be had right at the top so that they can sing about it and they can sort of disseminate their, their knowledge to the, the staff… There was often like that disconnect. (Professional 02, workshop)

Visible organisational commitment and strategic leadership was viewed as important to fostering a culture in which communication is prioritised and embedded through coordinated workforce development.

#### Theme 4.2: Knowledge, skills and confidence

This theme captures the wide spectrum of staff competence in both spoken communication and non-verbal skills, and how this proficiency (or lack thereof), directly impacted the quality of person-centred communication support.

#### Staff knowledge and skills

Variability in staff knowledge and communication competence was described as a key factor influencing the quality of communication support. This was often linked to the depth and quality of training staff received. In some cases, limited understanding led to misjudgements (for example, overestimating communication abilities) which resulted in ineffective support.

Some people have an amazing intuition and can adapt their language and delivery and they’re hypersensitive to how the person’s responding. Other people just charge ahead… without really picking up on the cues or understanding the person’s response (Professional 01, workshop)

Participants emphasised the need for staff to adapt their approach to suit individuals’ preferred communication styles, guided by the principle that *“communication is a human right”* (Professional 03). This included offering multiple means of expression and creating environments that support autonomy and understanding.

Adults with learning disabilities valued staff who were respectful, used humour, and supported decision-making using clear and accessible communication methods, such as visual timetables and simplified language.

And they [staff] try to explain things [to me] clearly sometimes… [they] try to understand what I was saying. (Adult with learning disabilities 06, workshop)

Conversely, communication breakdowns where staff spoke too quickly or staff perceived speech to be unintelligible were linked to frustration among adults with learning disabilities.

#### Understanding of non-verbal communication

Recognising and interpreting non-verbal communication was identified as essential for effective support, particularly for adults with severe communication needs. Staff who were skilled in reading subtle cues such as tone, gestures, and facial expressions were viewed as better equipped to provide responsive support.

**…** you see that support teams are so skilled at reading the sounds and the tones, and the body language, and the facial expressions. (Manager 03, workshop)

This view was also echoed by adults with learning disabilities who described the ability of staff to detect non-verbal cues that indicated changes in their mood.

They [staff] can also sense how you’re feeling without showing it… they can see… something’s not right… straight away with my body language, that I’m not okay. (Adult with learning disabilities 05, workshop)

However, some family carers raised concerns about inconsistent staff recognition of non-verbal signals, which they perceived as leading to distress or a loss of communication skills over time.

…she [my daughter] would tap on the settee… if [she] wanted to go to the toilet, and unfortunately some carers didn’t pick it up, so she gets annoyed and she stops. (Family carer 02, interview)

### Domain 5: Implementation process domain

#### Theme 5.1: Integrating communication strategies

This theme describes the process of trialling, evaluating, and embedding diverse communication strategies into daily routines to ensure they remain responsive to the user’s changing needs.

#### Trialling different strategies

Participants emphasised the importance of actively trialling and adapting communication strategies to align with the needs of individual adults with learning disabilities. Staff reported that effective use of tools such as AAC required flexibility and ongoing evaluation. As noted, *“flexibility [in staff’s communication approach] has to extend to that person’s communicative preference and their need.”* (Professional 09). Proactive adjustments, drawing on staff’s knowledge and understanding of available tools and strategies, were seen as essential to supporting adults with learning disabilities in both understanding and expressing their needs.

… [it] is making sure that, we give people the tools… ‘What tools have we tried? … have we tried environmental cues? Have we tried objects of reference? …have we tried less words? Have we tried pictures?’ (Manager 03, workshop)

These findings highlight the need for staff to approach communication support as a dynamic, responsive process. Embedding adaptive practices into routine interactions may enhance mutual understanding. This adaptation must be guided by person-specific feedback gathered collaboratively from the individual and those who know them best (e.g., family) to enhance mutual understanding.

#### ToC for improving the implementation of communication support for adults with severe learning disabilities in social care settings

**Fig. 1:**
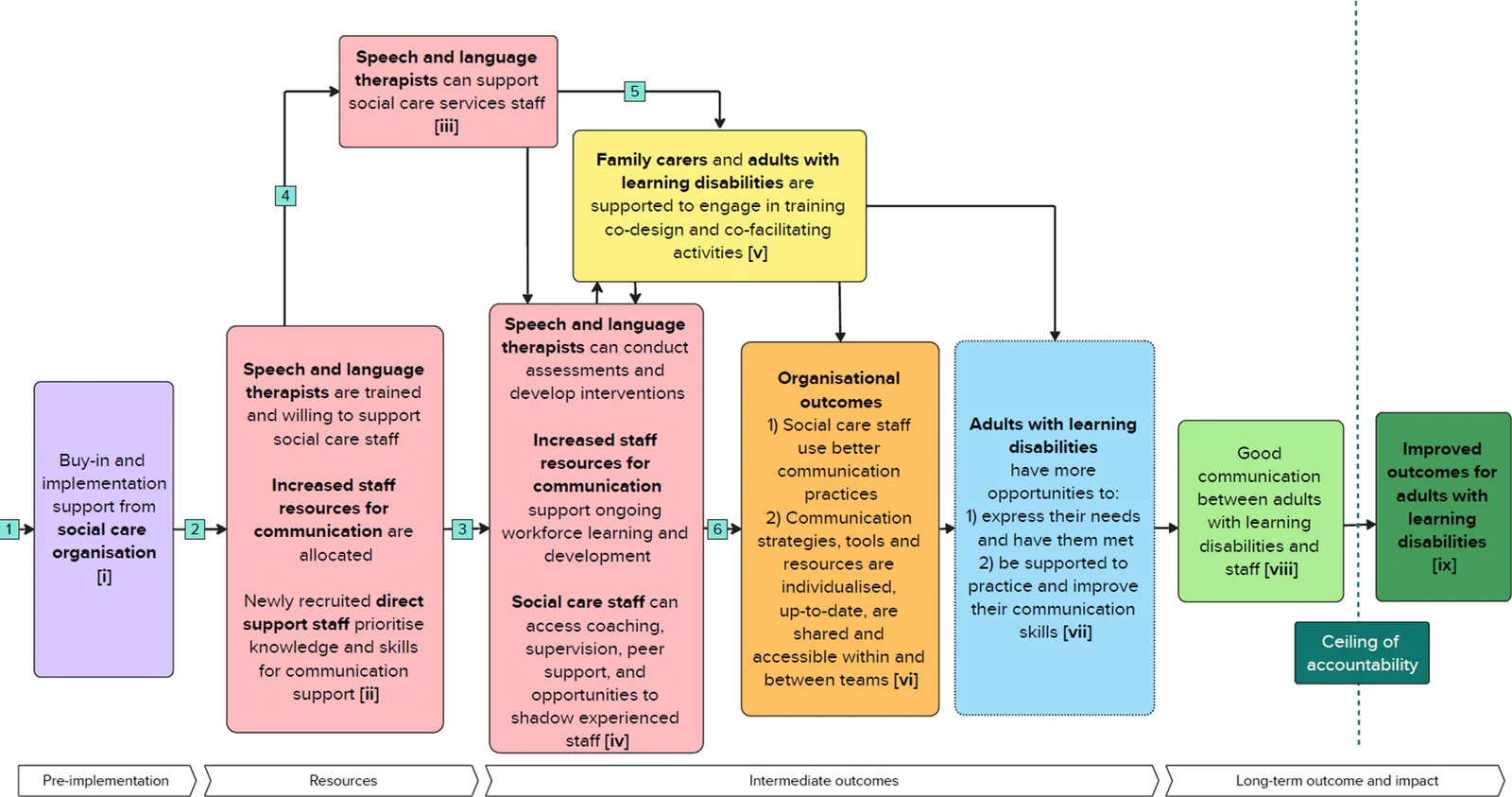
ToC for improving the implementation of communication support for adults with severe learning disabilities in social care settings

Figure 1 shows the ToC map developed with participants, specifying the activities required to achieve short-, medium-, and long-term outcomes and the desired long-term impact. The map also shows the necessary conditions that must be in place for causal pathways to lead to the impact (‘key assumptions’), and indicators to monitor the implementation of the interventions (See Table 7) [42]. See Additional Files 4 and 5 for a full version of the ToC map and legend. The findings from the analysis of stakeholders’ perceived barriers and facilitators to good communication support directly informed the development of the implementation-focused ToC. This ensured the map accurately captured the contextual and organisational factors affecting the implementation of communication support in social care settings.

**Table 7:** ToC outcomes and example indicators

Outcomes	Indicators
1) Collaboration with key decision makers; consultation activities with staff; resources assessment.	**[i]** Staff buy-in to interventions; staff knowledge of interventions aimed at improving the implementation on communication support; staff motivation to use communication support strategies and tools.
2) Coaching session(s) with speech and language therapists. Develop role description and competency assessment. Advisory documents: job description and competence assessment; job shadowing.	**[ii]** SLT assessment of perceived knowledge and skills; direct support perceptions of SLT’s knowledge and skills in improving staff communication support. Workforce data: number of staff appointed; staff retention levels. Staff receive feedback on communication practices. Staff shadowing opportunities.
3) Coaching session(s) with speech and language therapists. Core training for staff in roles that increase resources for communication. Resources assessment; in-situ practice sessions. Supervision and mentorship; peer support sessions.	**[iii]** Staff competence measures; speech and language therapist involvement; team feedback on the effectiveness and impact of speech and language therapist support; training statistics; training matrix and competency checks; protected time for training; Staff feedback on communication practices.
4) Coaching session(s) with speech and language therapists.	**[iv]** Staff competence measures.
5) Collaboration activities with family carers and adults with learning disabilities.	**[v]** Frequency of communication with family carers; Whether feedback from families and adults with learning disabilities has informed the training and supervision development.
6) Core training for staff in roles that increase resources for communication. Coaching; learning needs assessment; supervision. Process assessment and advisory documents: information recording and sharing; skills coaching. Resource development coaching collaboration activities with family carers and adults with learning disabilities; resource identification.	**[vi]** Quality of communication documentation (e.g. verbal handovers, assessments); staff attendance at handover meetings: Evidence of staff engagement with handover documents.
	**[vii]** The frequency adults with learning disabilities can express themselves, have their needs met, and practise their communication skills with staff. Audit of staff evaluation and adaption of communication strategies.
	**[viii]** Observe communication between adults with learning disabilities and staff, including the strategies and tools used and the level of engagement achieved. Staff appraisals on communication support practices. Staff self- and peer evaluation on use of communication interventions.
	**[ix]** Whether the communication support needs of adults with learning disabilities are identified and appropriately addressed by support staff. Adults with learning disabilities’ satisfaction with communication support. Levels of engagement and participation in activities by adults with learning disabilities.

Improved outcomes for adults with learning disabilities, identified by participants as the overarching impact of the ToC, provided the foundation for understanding how change could be achieved. Meaningful change was viewed as contingent on early organisational buy-in, leadership commitment, collaboration with key decision-makers, and adequate resourcing during pre-implementation. Achieving intermediate outcomes, such as developing effective, person-centred support that strengthens communication partnerships, was identified as essential through three pathways: (1) collaborative development of communication resources and training, (2) improved information sharing and recording processes, and (3) ongoing skills development, peer mentoring, and supervision. The successful execution of these pathways is dependent on several key assumptions, primarily relating to the availability of resources and the sustained willingness and capacity of stakeholders to consistently and effectively engage with improvements to the implementation of good communication support for adults with severe learning disabilities. Assumptions underpinning the ToC related to resource availability, workforce capacity, prioritisation of communication support, and organisational commitment, all of which pose risks in under-resourced social care settings. Participants also contributed to identifying indicators for evaluating implementation processes and outcomes. See Additional File 6 for a detailed report on the ToC components.

## Discussion

In this study, we aimed to explore the barriers and facilitators perceived by stakeholders in providing good communication support to adults with learning disabilities within residential social care settings. Furthermore, we sought to identify the key features and intervention components stakeholders considered essential for improving implementation. Beyond identifying these individual elements, the study utilised a ToC framework to map key pathways and underlying mechanisms through which these components interact. By mapping the causal chain between practice change and intended outcomes, we aimed to provide a comprehensive understanding of the systemic and individual processes required to sustain good communication support for adults with severe learning disabilities.

Though many of the barriers and facilitators identified in this paper echo findings from previous implementation research, this study advances understanding by examining how these factors interact within the specific context of communication support in social care for adults with severe communication needs. By situating these known factors within a ToC framework, this paper emphasises key mechanisms through which organisational and individual conditions influence the implementation of good communication support, an area that has received limited attention in the literature.

Using the ToC in collaboration with stakeholders, we identified short-, medium-, and long-term outcomes that were considered important for improving the implementation of good communication support for adults with severe learning disabilities. The findings, categorised via the CFIR framework, directly informed the development of the ToC map by defining assumptions, causal pathways and feedback loops. For example, participants highlighted that limited financial resources and the restricted availability of specialist SLT expertise acted as significant ‘Outer Setting’ barriers to sustained implementation of good communication support. To mitigate these, the ToC map incorporates specific mechanisms such as enhanced information-sharing systems and the formalisation of ‘increased staff resources for communication’ as critical drivers to bridge the gap between specialist guidance and delivery. These findings highlight how organisational commitment and the accessibility of tailored resources (identified as ‘Inner Setting’ facilitators) serve as the necessary preconditions for the causal chain leading to consistent and person-centred communication support. By mapping these CFIR-derived insights onto the ToC, we illustrate how systemic shifts in leadership and resource allocation are required to move from fragmented interactions toward sustained implementation.

The use of the CFIR informed a comprehensive analysis of core contextual, organisational and individual-level barriers and facilitators, as perceived by stakeholders, in achieving effective communication support. Collectively, these findings advance current knowledge by providing a system-level and relational understanding of communication support implementation within social care settings.

### What does this paper add?

This paper contributes a new implementation-focused ToC that integrates existing evidence on organisational and individual mechanisms of practice change in communication support for adults with learning disabilities. The ToC outlines how pre-implementation processes such as leadership commitment, and readiness influence the delivery and sustainability of communication support interventions [47], providing theoretically grounded framework for translating known facilitators into practical, context-specific implementation strategies.

Consistent with previous research [48, 49] organisational and managerial leadership in resource allocation, collaborative intervention development and implementation, and a sustained commitment to promoting change are necessary for improving staff communication practices and organisational processes. To support these activities, we note the need for competency and values-based recruitment strategies to appoint staff with a readiness and an awareness of the knowledge needed for high-quality communication support. Employers are aware of these recruitment strategies; however, they struggle to implement them effectively [50], emphasising the need for strategic organisational-level planning to align recruitment strategies with service needs.

This paper also highlights the critical role of specialist professionals within social services workforce models. While SLTs provide essential training, modelling, and quality assurance for good communication support [31], resource limitations restrict their capacity for ongoing input, reflecting prominent outer setting domain barriers [51, 52]. Participants noted the potential benefit of additional trained staff, such as practice leaders working under the supervision of SLTs to deliver real-time coaching, feedback and ensuring consistency in practice. The ToC assumptions further indicate that capacity-building activities should be considered to ensure SLTs and social care staff have the competency and readiness to effectively delegate, and implement tasks according to individual and service needs, strengthening the training and supervision required for effective staff communication practice. The principles outlined in this ToC align well with the task-sharing approach advocated in global health [53].

At the staff level, this paper highlights the significance of knowledge, skills, and responsiveness to individual communication preferences and needs, including recognising and supporting non-verbal communication, particularly for adults with learning disabilities with little or no speech. While the use of AAC interventions have been demonstrated to benefit communication interactions [54], our findings add to the literature on the persistent barriers to embedding these practices into everyday interactions, despite widespread awareness of their benefits [11]. Consistent with previous research [55], we provide further evidence that effective communication support requires both organisational enablers (e.g. mentoring, modelling, peer learning) and collaborating with family carers and adults with learning disabilities in co-designing approaches that are responsive to individual communication preferences. Whilst our conversations were about good support for communication more broadly, many of the group members focused on AAC. We recognise that many people with learning disabilities do not use symbolic forms of communication and rely more on communication partners to interpret their needs. We also recognise that communication is broader than just forms of communication.

This paper also contributes to the limited literature on how communication support outcomes are measured [56, 57] by identifying outcomes and indicators with diverse stakeholders to guide evaluation, including measures of engagement, staff responsiveness to the communication needs and preferences of adults with learning disabilities, and auditing record keeping and information sharing systems. Existing studies have largely described barriers and facilitators [58, 59]; however, few studies have examined how these factors interact to form implementation processes. The ToC developed here makes these causal pathways explicit, integrating stakeholder insights with implementation theory to show where change efforts should be directed.

It is noteworthy, that stakeholder discussions of barriers and facilitators predominantly centred on communication methods and observable interaction features, such as the use of AAC, non-verbal cues, and information-sharing systems, with limited attention to underlying language structures such as vocabulary complexity, or language processing. This pattern likely reflects the practical, task-orientated priorities of direct support staff and the wider social care workforce. However, it may also indicate a gap in workforce understanding of how linguistic complexity affects comprehension, potentially leading to overestimation of what adults with severe learning disabilities understand from spoken or written information, as highlighted in our findings in the ‘Individuals’ domain.

### Strengths and limitations

Bringing together diverse stakeholder perspectives, with opportunities for critical reflection, enhanced the relevance, acceptability and inclusivity of the potential or planned implementation intervention model. The combined use of the ToC and the CFIR added rigour and transparency by clarifying how theoretical assumptions informed the design and analysis. The ToC map further illustrated programme logic, supporting the plausibility of intervention pathways. Additionally, identifying stakeholder-prioritised features for improving communication support for adults with severe learning disabilities, alongside a detailed examination of the social care context, enabled a systems-oriented understanding of both barriers and facilitators to practice change. This is critical in social care research as it increases the likelihood that interventions will be both feasible and sustainable in real-world settings [43].

A key consideration in our analysis was ensuring attention to the perspectives of adults with learning disabilities, all of whom communicated using speech. We acknowledge that the input of adults with learning disabilities is represented by fewer direct quotes than other stakeholders. However, this reflects our careful interpretive approach and commitment to avoiding over-interpretation of the data. We were mindful to avoid ‘forcing inclusivity’ in our findings, where data from adults with learning disabilities did not clearly align with the implementation-focused ToC components and communication support. Throughout all study stages, reflective team discussions guided our interpretation of how inclusivity was genuinely represented in the findings. This caution, while resulting in fewer direct quotes, reflects the inherent complexity of capturing and interpreting contributions from this group within the study’s design.

The workshop structure also created practical limitations. The division of workshops into four distinct topics challenged participant consistency, as some stakeholders only attended single sessions, which limited their familiarity with the iterative development of the ToC map. The complexity of condensing data into an accessible, non-technical format within brief sessions meant that a full, final version of the map could not be collaboratively reviewed in its entirety. We acknowledge that some elements may have been overlooked and so we invited participants to provide additional input, including on the dissemination of study findings. Despite efforts to recruit widely, the sample was predominantly White British women. In addition, there was variability in workshop attendance, ranging between 25% and 100% per session. Attendance was driven by both late-stage recruitment and participant availability. While overall retention remained stable across the study timeline, the variability in ToC workshop attendance resulted in reduced representation from some groups, such as family carers. This may have influenced the depth of the collective reflection.

We also acknowledge that the experiences of adults with mild versus severe learning disabilities are likely to differ, as will approaches to gain views and experiences. In this study, we included people with mild learning disabilities in our groups. We also included family carers of people with severe to profound intellectual disabilities so that the views and experiences of people with severe to profound learning disabilities were represented in our Theory of Change. Ensuring robust training and support for staff in roles that facilitate such activities will be crucial to maintaining the quality and inclusivity of future intervention development.

### Implications for practice and policy

Improving communication support in social care settings requires sustained organisational commitment, with providers embedding communication support within service priorities and ensuring that training is underpinned by clear implementation strategies, ongoing monitoring, and access to appropriate resources. Building capacity within teams is also essential. Our findings indicate staff in roles providing additional communication support, alongside service managers and SLTs, could provide coaching feedback and practice oversight, helping to maintain workforce skills and fidelity, particularly in contexts where specialist communication resources are scarce.

The ToC also highlights the value of collaboration with people with lived experience and across professional teams, with co-produced implementation strategies ensuring communication support is person-centred, contextually appropriate, and acceptable to both adults with learning disabilities and staff. Workforce development policies should prioritise values- and competency-based recruitment to ensure staff readiness for delivering effective communication support.

Although the Accessible Information Standard sets expectations for communication support in England, provision remains inconsistent across adult social care services. Embedding inclusive communication within statutory guidance, commissioning standards, and workforce training could strengthen person-centred practice and contribute to improved outcomes for people with severe and profound learning disabilities.

## Conclusion

Our findings suggest that effective communication support in social care requires more than staff training alone. It is likely to depend on organisational commitment, leadership engagement, and collaborative workforce models. Using the ToC, Template Analysis, and CFIR frameworks, we identified implementation processes that are likely to be essential for sustainable change in often resource-constrained social care settings. Our paper contributes a theoretically grounded, implementation-focused model and a framework for evaluating implementation quality and efficiency. Embedding co-production activities, capacity-building, and organisational alignment with the communication rights of adults with learning disabilities are fundamental to achieving equitable and effective practice in social care settings.

## Supplementary Information

Below is the link to the electronic supplementary material.

Supplementary Material 1

Supplementary Material 2

Supplementary Material 3

Supplementary Material 4

Supplementary Material 5

Supplementary Material 6

## Data Availability

The datasets generated and analysed during the current study are not publicly available due to the need to protect the anonymity and confidentiality of the participants, which was a fundamental ethical requirement of the study and a condition of their consent.

## Abbreviations

AAC: Augmentative and alternative communication
CFIR: Consolidated Framework for Implementation Research
ICALD: Improving Communication with Adults with Learning Disabilities
SLT: Speech and language therapist
ToC: Theory of Change
