# Supplementary material for: Developing a theory of change to improve the implementation of communication support for adults with severe learning disabilities in social care settings
Source: BMC Health Serv Res. 2026 Aug 6;26:1518. doi: 10.1186/s12913-026-15154-1 (PMC13733076; doi:10.1186/s12913-026-15154-1)
Supplement: Supplementary file 1 [file 12913_2026_15154_MOESM4_ESM.pdf]

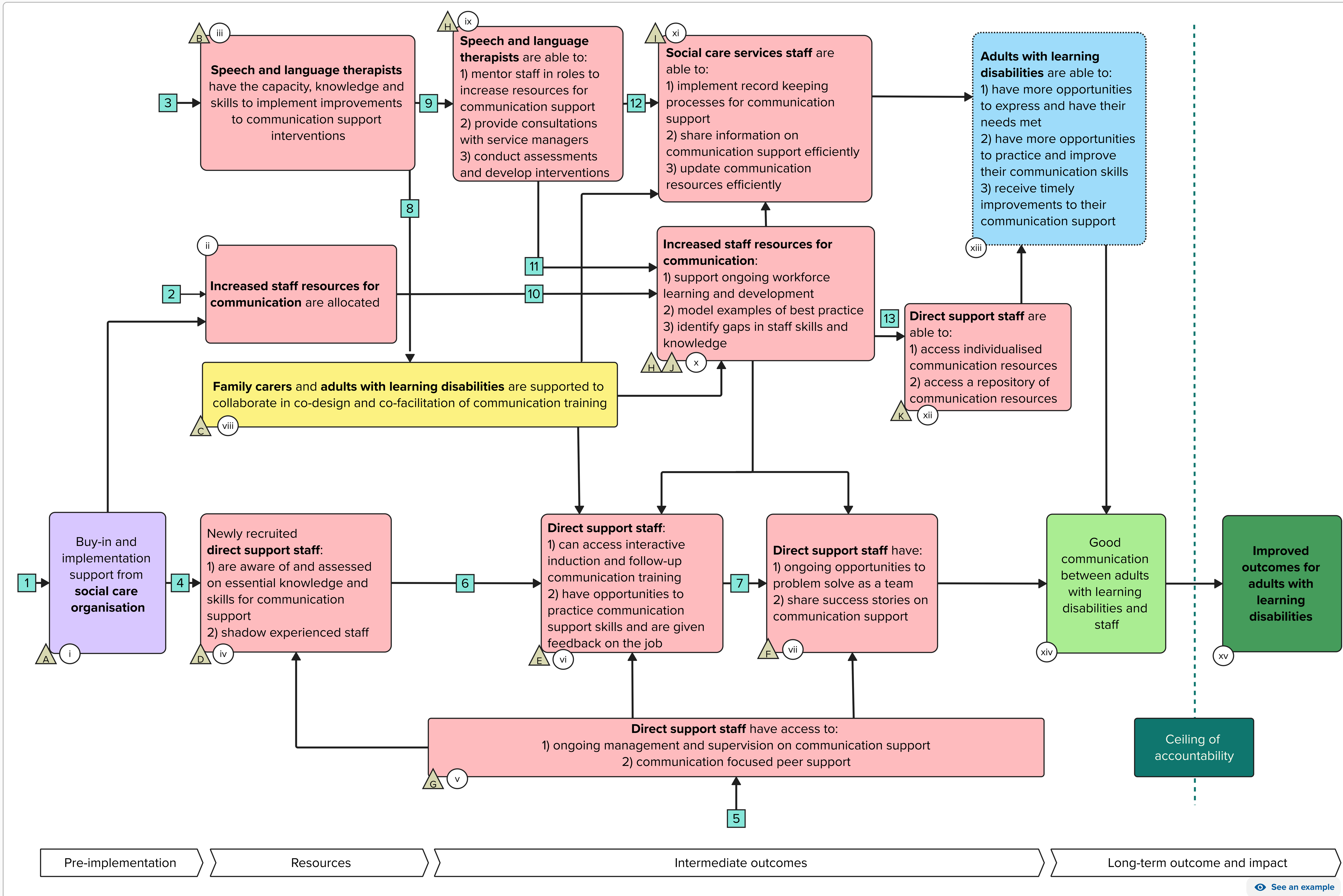
Speech and language therapists have the capacity, knowledge and skills to implement improvements to communication support interventions
Speech and language therapists are able to: 1) mentor staff in roles to increase resources for communication support 2) provide consultations with service managers 3) conduct assessments and develop interventions
Social care services staff are able to: 1) implement record keeping processes for communication support 2) share information on communication support efficiently 3) update communication resources efficiently
Adults with learning disabilities are able to: 1) have more opportunities to express and have their needs met 2) have more opportunities to practice and improve their communication skills 3) receive timely improvements to their communication support
Increased staff resources for communication are allocated
Increased staff resources for communication: 1) support ongoing workforce learning and development 2) model examples of best practice 3) identify gaps in staff skills and knowledge
Direct support staff are able to: 1) access individualised communication resources 2) access a repository of communication resources
Family carers and adults with learning disabilities are supported to collaborate in co-design and co-facilitation of communication training
Buy-in and implementation support from social care organisation
Newly recruited direct support staff: 1) are aware of and assessed on essential knowledge and skills for communication support 2) shadow experienced staff
Direct support staff: 1) can access interactive induction and follow-up communication training 2) have opportunities to practice communication support skills and are given feedback on the job
Direct support staff have: 1) ongoing opportunities to problem solve as a team 2) share success stories on communication support
Good communication between adults with learning disabilities and staff
Improved outcomes for adults with learning disabilities
Direct support staff have access to: 1) ongoing management and supervision on communication support 2) communication focused peer support
Ceiling of accountability
Pre-implementation
Resources
Intermediate outcomes
Long-term outcome and impact
See an example
