# Supplementary material for: Developing a theory of change to improve the implementation of communication support for adults with severe learning disabilities in social care settings
Source: BMC Health Serv Res. 2026 Aug 6;26:1518. doi: 10.1186/s12913-026-15154-1 (PMC13733076; doi:10.1186/s12913-026-15154-1)
Supplement: Supplementary file 2 [file 12913_2026_15154_MOESM1_ESM.docx]

**Additional File 1: Topic Guide**

# **Theory of Change workshops and interview topic guide**

The guidance includes topics that will be covered during the course of the study in the workshops, interviews, and supported conversations. The topics won’t necessarily be covered in the order that they appear in this guide.

## Introduction

- Introductions; thank participant(s) for their time and involvement in the study; timeframe of workshop/interview; time and duration of scheduled break during workshop/interview; offer refreshments to participants.
- Participants will be reimbursed for their time and contribution to the study.
- Explanation of the study:
  - Description of communication and how we will define the Theory of Change framework in this study
  - The aim of this study is to understand what outcomes people would like to see in social care services in relation to better communication support. We are also interested in better understanding ideal practices and the barriers and facilitators faced by support staff in providing consistent high-quality communication support to adults with learning disabilities (LD).
  - The workshop/interview discussions are focussed on your personal experiences of these topics. There are no wrong or right answers and please feel free to share as much or as little you are comfortable with talking about.
- Confidentiality:
  - Within the workshop/interview, safe storage and transfer of data (audio recording and personal data)
- By agreeing to take part in this study, you also consent for the workshop/interview to be audio recorded.
- All participation is voluntary:
  - Participants are free to have a break or end the workshop/interview at any point without giving a reason.
  - At the end of the workshop/interview, we will ask you if you would be happy to be invited to another workshop/interview to continue discussions on how communication support for people with LD can be improved in social care settings.
- Do you have any questions that would you like to ask before beginning the workshop/interview? [answer questions]
- Ok, are you ready to start?
- I will start the recording now and I will let you know when I stop recording.

## **Topic 1:**

## Goal setting

We are looking to better understand the barriers and facilitators faced by support staff in providing consistent high-quality communication support to adults with learning disabilities living in residential social care. To guide the focus of this study, we would also like to better understand what outcomes people would like to see in social care to inform the development of training and post-training packages for staff to support communication strategies of adults with LD in social care settings.

- In your opinion, what should be the goal of the intervention? *(impact)*

*Prompts:*

- What should communication support look like?
- What is important about communication strategies in social care settings?
- How do you think staff should be involved in supporting communication?
- Is the goal achievable?
- Is everyone happy with the goal?

## Barriers and facilitators to practice change

- What factors help or create barriers to improving communication support for adults with learning disabilities in social care settings?

*Prompts:*

- Individual factors: staff skills, knowledge, confidence; social influences (managerial influence, other colleagues, family carers, social pressures)
- Organisational factors: workplace environment; availability of resources, organisational structure
- In your opinion, what factors could support the ongoing implementation of new approaches to supporting the communication of adults with learning disabilities in social care settings?

## **Topic 2:**

## Implementation context

## What are the conditions necessary for successful implementation of communication support?

## How do current workforce practices or routines impact how communication skills are used?

## Staff skills and knowledge

- In your opinion, what skills and knowledge are important for supporting the communication of adults with communication impairments?

*Prompts:*

- What do you understand about supporting the communication of this group?
- What are the main goals in supporting the communication of adults with LD?
- Are there guidelines or knowledge from staff training that inform current practice?
- Can you give examples of skills and/or training that help or limit the support staff provide around the communication of the needs of adults with LD?

Long-term goal

- What is the desired long-term goal for this study in relation to communication support for adults with learning disabilities?

## **Topic 3:**

## Ideal practice in training and delivery of communication support

- In your opinion, what do you consider to be best practice in the day-to-day delivery of supportive communication for adults with adults impaired communication?

*Prompts:*

- What strategies are currently used to support people with LD to express their needs/makes choices/understand information? Why are these strategies helpful/unhelpful? How can you tell this?
- Can you provide examples of what supportive communication looks like? When does this happen? And how often?
- Are there strategies that have not been mentioned/you have not experienced that you feel would be helpful to improve communication support for this group?
- Do you feel that these examples of best practice are always achievable?

Preconditions, and causal pathways for communication support

- What are the necessary outcomes (preconditions) required to successfully improve the implementation of communication support for adults with severe learning disabilities?

*Prompt:*

- What immediate changes do we need to see directly after [outcome]? (*short-, intermediate-, and long-term outcomes*)
- What specific activities are required to achieve these outcomes along each step of the way?

*Prompt:*

- What practical training, post-training packages, or management actions are needed to drive change?

**Topic 4:**

Assumptions and measuring change

- What assumptions and workforce knowledge gaps need to be addressed to achieve our long-term outcomes?
- What are our key indicators of change, and how can we check if our approach has made things better?

*Prompts:*

- What do you personally want to change in communication support practice? (*What I want to change*)
- What specific outcomes or milestones will we measure? (*What we will measure*)

How exactly will we measure what will change following the training? (*How will we measure what will change*)

# **Supported conversations with adults with learning disabilities topic guide**

## Introduction

- Introductions; thank participant for their time and involvement in the study; timeframe of conversation; breaks can be taken when needed during the supported conversation; offer refreshments to participant.
- Participant will be reimbursed for their time and contribution to the study.
- Explanation of the study:
  - Description of communication and how we will define the Theory of Change framework
  - We want to understand how to improve communication for people like you that live in social care. Today we will talk about your experiences.
    - There are no wrong or right answers, and you can talk about as much or as little you are comfortable with talking about.
- Confidentiality:
  - Safe storage and transfer of data (audio recording and personal data)
- By agreeing to take part in this study, you also consent for the workshop/interview to be audio recorded.
- All participation is voluntary:
  - Participants are free to pause or end the conversation at any point without giving a reason.
- Do you have any questions that would you like to ask before beginning our conversation? [answer questions]
- Ok, are you ready to start?
- I will start the recording now and I will let you know when I stop recording.

1. What does good communication support mean to you?
2. Do staff help you to communicate/understand information/make choices?

*Prompts:*

- What do they do?
- Do they always do this?

3. What is good about how staff support your communication?
4. What is unhelpful about how staff support your communication?

We also want to make a training program for staff that work in social care to improve how they support your communication needs.

5. What things would help staff and you understand each other better?

*Prompt*:

What is important to you about communicating your needs/feelings/choices?

**Stakeholder focus group topic guide**

Interventions

- Have you heard of [name of intervention] before?
- When have you used it?
- Did you receive training on how to use it?
  - if yes: from who?
  - Was it easy/difficult to implement?
- Can you provide an example of when you have used [name of intervention]?
  - Did you find using [name of intervention
  - ] helpful/unhelping in supporting communication?
  - if yes: can you share how it has helped? What features of the intervention were beneficial and for whom?
- What makes it difficult/easy to use [name of intervention]?
  - *Prompts:* contextual or environmental factors, individual factors (confidence in implementing the intervention, knowledge), system level factors (organisational policies, workplace culture)
- Are there any interventions that we have missed that have help to support communication?
- Is this an intervention you have used yourself or observed someone else using?

Measures

- Are you familiar with [name of measure]?
  - If yes: when have you used it?
- Do you feel it is an appropriate measure to assess [behaviour]?
  - Do you feel the measure captures what it has been designed to do?
  - Is something missing?
  - Discuss:
    - burden of time needed to complete the measure,
    - frequency to administer the measure,
    - skills/training/knowledge needed to score the measure/conduct the observation etc,
    - 'patient' rated versus 'clinician rated outcome measures – are they objective?
- Do you have suggestions for measures that would be helpful for family carers/ adults with learning disabilities/ professionals that have been missed?
  - Why do you think these would be helpful?
- Are there any measures that we have missed that have help to support communication?
- Have you used or observed someone else using a questionnaire/ conducting an assessment/observation
  - What problem was [name of measure] trying to identify/measure/identify?
