# Supplementary material for: Developing a theory of change to improve the implementation of communication support for adults with severe learning disabilities in social care settings
Source: BMC Health Serv Res. 2026 Aug 6;26:1518. doi: 10.1186/s12913-026-15154-1 (PMC13733076; doi:10.1186/s12913-026-15154-1)
Supplement: Supplementary file 3 [file 12913_2026_15154_MOESM2_ESM.docx]

**Additional File** **2** - Methods

**Table 1S.** Organisation, sector and professional roles of staff participants.

|  | **Overall**  **(*n* = 29)** |
| --- | --- |
| **Organisation/sector**  Non-profit/charity  Public Sector (NHS, Local Authority, commissioning)  Private specialist health and social care  **Professional roles***  Co-production and best practice lead  Integrated Commissioning Manager  Occupational therapist  Practice lead  Social worker  Speech and language therapist  Team manager  Trainee speech and language therapist apprentice  Service managers  Support workers  Direct support staff  Lead senior support worker  Trainers  Area trainer  Trainer | 13 (44.8%)  8 (27.6%)  8 (27.6%)  1 (3.4%)  1 (3.4%)  1 (3.4%)  1 (3.4%)  1 (3.4%)  7 (24.1%)  1 (3.4%)  1 (3.4%)  5 (17.2%)  7 (24.1%)  1 (3.4%)  1 (3.4%)  1 (3.4%) |

* Professional roles as defined by participants.

Researcher positionality

The ICALD programme was conceived with the belief that individuals with learning disabilities can receive effective communication support in social care settings when staff are well-trained and supported. Initial discussions on the project idea were held with the Autistica Network and Insight Group, as part of Autistica Specialist Interest Group on Language and Communication, 2018-2022. The group was led by VS and included two ICALD research co-applicants (J.B. and K.L.). While there is long-standing recognition of what good communication support should look like, there is limited evidence on how best to implement this effectively in practice. Having a clear emphasis on communication partnerships was central to this study, with the understanding that meaningful interactions are closely linked to staff being able to recognise, interpret, and respond to individuals’ communication needs and preference. This informed the focus on the implementation of staff training and the organisational conditions that enable skills to be translated effectively into everyday practice.

**Table 2S.** Theory of Change terminology and definitions.

| **Terminology** | **Definition** |
| --- | --- |
| Impact | The highest-level change the program ultimately aims to contribute to, but for which it cannot be held solely accountable. |
| Long-term outcome | The final outcome of the program once activities and intermediate outcomes have been achieved. |
| Pre-condition (short-term, intermediate  and long-term outcomes) | The anticipated results of the interventions - conditions that are not currently in place but must be established to maintain the causal pathway and ultimately achieve the intended impact. |
| Ceiling of accountability | The point in the causal pathway beyond which indicators are no longer used to assess progress and the programme is no longer held responsible for achieving outcomes. This threshold is typically positioned between the long-term outcome and the overall impact. |
| Indicator | Thing that can be measured to determine progress in working towards or achieving each outcome. |
| Interventions | The components of the complex intervention. |
| Assumptions | External conditions which lie beyond the control of the project but must exist for each outcome to be achieved. |

Adapted from De Silva and colleagues (2014)
