# Supplementary material for: Developing a theory of change to improve the implementation of communication support for adults with severe learning disabilities in social care settings
Source: BMC Health Serv Res. 2026 Aug 6;26:1518. doi: 10.1186/s12913-026-15154-1 (PMC13733076; doi:10.1186/s12913-026-15154-1)
Supplement: Supplementary file 4 [file 12913_2026_15154_MOESM5_ESM.docx]

**Additional File 5. Full ToC Legend**

# Theory of Change Legends

## Interventions – components and function

| **Number** | **Components** | **Function** |
| --- | --- | --- |
| 1 | Collaboration with key decision makers; consultation activities with staff; resources assessment. | Collaborate with high-level organisation leads, service managers and frontline to co-develop activities; negotiate relevant resources with high-level organisation leads and service managers. |
| 2 | Develop role description and competency assessment. | Increased staff resources for supporting communication support are identified. |
| 3 | Coaching session(s) with speech and language therapists. | Develop the knowledge and skills of speech and language therapists necessary to support social services staff and collaborate with stakeholders during activities. |
| 4 | Advisory documents: job description and competency assessment; job shadowing. | Provide guidance on modifications to frontline staff job descriptions and person specifications; promote the assessment of frontline staff competency in supporting complex communication needs during recruitment; identify and implement best practices in shadowing processes (for new frontline staff). |
| 5 | Supervision and mentorship; peer support sessions. | Identify and implement best practices for ongoing staff support (supervision) and peer support and promote their uptake in services. |
| 6 | Resources assessment; in-situ practice sessions. | Consult with service managers to identify protected time, space, and access to resources for training; identify and integrate processes that increase opportunities to practice skills. |
| 7 | Case reviews; reflective practice sessions; staff awareness-raising sessions. | Identify processes for case reviews for adults with learning disabilities and offer guidance and structure on how case reviews can be implemented and embedded within existing staff meetings; engage with service managers and frontline staff to promote motivation for communication support and the sharing of lessons learned. |
| 8 | Collaboration activities with family carers and adults with learning disabilities. | Collaborate with adults with learning disabilities and family carers in co-designing and co-facilitating activities for induction and follow-up communication training (individualised and service-specific). |
| 9 | Coaching session(s) with speech and language therapists. | Provide guidance for speech and language therapists on how to support service managers, direct support staff and mentor staff in roles to improve the implementation of communication support. |
| 10 | Core training for staff in roles that increase resources for communication. | Provide relevant communication training. |
| 11 | Coaching; learning needs assessment; supervision. | Provide training on modelling communication strategies correctly; identify and address staff learning needs; offer supervision from speech and language therapists. |
| 12 | Process assessment and advisory documents: information recording and sharing; skills coaching. | Provide guidance for developing up-to-date individualised communication strategies; develop and integrate best practice procedures for accurate information sharing and recording within services regarding communication support. |
| 13 | Resource development coaching; collaboration activities with family carers and adults with learning disabilities; resource identification. | Provide guidance on training and resources needed to support the development of individualised communication strategies; collaborate with family carers in the process of updating communication strategies; identify sources of accessible communication resources for adults with learning disabilities. |

## Theory of Change outcomes, assumptions and indicators

| **Outcome** | **Assumption** | **Indicators** |
| --- | --- | --- |
| Buy-in and implementation support from **social care organisation.** | A. High-level organisational leaders are willing to provide resources; social care service staff are willing to prioritise activities to improve good communication support. | **[i] Staff interviews (buy-in to interventions):**   - Explore staff willingness and openness to implement changes to improve communication support. - Assess understanding and application of interventions during recruitment and training.   **In-depth interviews and observations (direct support staff):**   - Determine staff knowledge of interventions aimed at improving the implementation on communication support. - Identify whether staff report using the intervention and how to implement it.   **Anonymous questionnaires (social care services staff):**   - Staff motivation to use communication support strategies and tools.   **Staff meetings:**  Focus: feedback on support for communication improvement:   - Assess whether service managers actively gather and respond to staff feedback (e.g. “You said, we did”). |
| Newly recruited **direct support staff**:   1) are aware of and assessed on essential knowledge and skills for communication support 2) shadow experienced staff | D. Service managers and organisations have the resources, willingness and capacity to amend their recruitment processes. | **[iv] Recruitment materials** include communication support knowledge and skills as selection criterion.  **Team meetings and supervision:**   - Staff receive feedback on communication practices. - Method: anonymous feedback forms/ minutes of supervision records/ observations of meetings.   **Process measure and interviews:**   - Assess whether all staff have opportunities to shadow experienced staff. |
| **Direct support staff**:   1) can access interactive induction and follow-up communication training 2) have opportunities to practice communication support skills and are given feedback on the job | E. Direct support staff are willing to engage in Continued Professional Development; they are motivated and have the time and space at work to access training; knowledge and skills from communication training impacts staff practice. | **[vi] Training statistics (percentages/counts):**   - Frequency of induction and follow-up training sessions offered per year. - Number and percentage of staff attending follow-up training. - Manager attendance at follow-up training sessions.   **Staff interviews**  Focus: coaching and skills review   - To assess how well communication skills were acquired and applied after initial training.   **Training matrix and competency checks:**   - Used to identify how often training is needed within the service. - Tracks training delivery, frequency, and alignment with staff needs to support adults with learning disabilities.   **Protected time for training:**   - Amount of time allocated by the organisation for staff and managers to attend training.   Whether follow-up training is:   - Advertised in a timely manner and accessible way. - Held in accessible locations - Supported by protected time for staff to attend.   **Feedback on communication practices:**  Staff receive communication focused feedback through:   - Team meetings. - Supervision sessions. - Peer mentoring and training opportunities. |
| **Direct support staff** have:   1) ongoing opportunities to problem solve as a team 2) share success stories on communication support | F. Direct support staff will utilise and implement communication strategies consistently; direct support staff and service managers are willing to address whether strategies are implemented, consistently, and accurately, and what strategies are difficult to implement and why. | **[vii] Audit areas:**  **1. Training and supervision**   - Frequency of training and supervision availability. - Satisfaction with training and supervision among social care staff, speech and language therapists, and other professionals involved in interventions aimed at improving the implementation on communication support. - Methods: Questionnaires, interviews, focus groups.   **2. Staff confidence**   - Confidence levels in communicating with adults with learning disabilities - Method: Questionnaire   **3. Training quality:**   - Feedback on communication training. - Method: Training feedback forms.     **4. Practice and implementation:**   - Quarterly review of staff demonstration of good practice.   Feedback from adults and family carers on current communication support.   - Method: Questionnaires, interviews   Assess staff implementation of core communication strategies   - Method: Observation   Opportunities for reflection and improvement:   - Availability of processes for staff to reflect on challenges and make improvements. - Methods: Surveys, observations. |
| **Direct support staff** have access to:   1) ongoing management and supervision on communication support 2) communication focused peer support | G. Service managers have established systems to support new and existing direct staff to develop and apply their learning. | **[v] Audit areas:**  **Management, supervision, and peer support systems:**   - Identify what formal systems are in place to support staff.   **Staff perspectives:**   - Assess staff access to and experience with supervision and peer support, including barriers and facilitators. - Methods: Questionnaires, interviews, or focus groups. |
| **Direct support staff** are able to:   1) access individualised communication resources 2) access a repository of communication resources | K. Direct support staff can access appropriate individualised communication strategies and a repository of resources. | **[xii] Audit areas:**  Record where core and individualised communication strategies/aids are stored and which are readily accessible to staff.   - Method: checklist.   Monitor whether staff have read and reviewed the communication strategies.   - Method: Sign-in sheet. |
| **Family carers** and **adults with learning disabilities** are supported to collaborate in co-design and co-facilitation of communication training. | C. Adults with learning disabilities and family are available and willing to develop and co-facilitate training activities (individualised and service-specific); The ICALD team can recruit adults with learning disabilities who can be supported contribute and collaborate in developing training activities. | **[viii] Frequency of communication with family carers:**   - Assess how often conversations occur regarding the development of both general and individualised communication strategies.   **Family survey and interview objective:**   - Gather feedback on desired changes to supervision and training.   **Audit:**   - Assess whether feedback from families and adults with learning disabilities has informed the training and supervision development. - Evaluate the processes supporting family carer involvement in training needs analysis. - Review the impact of their input on improving communication support standards. |
| **Increased staff resources for communication** are allocated. |  | **[ii] Workforce Data:**  **Number of staff appointed:**   - Includes direct support staff, speech and language therapists, and practice leaders.   **Staff Retention Levels:**   - Track retention rates for each role over a defined period (e.g., annually). |
| **Increased staff resources for communication**:   1) support ongoing workforce learning and development 2) model examples of best practice 3) identify gaps in staff skills and knowledge | H. Speech and language therapists are available and willing to provide mentoring and consultancy support. J. Funding for training is available. | **[x] Assessment and consultancy:**  **Speech and language therapist involvement.**   - Track the frequency of speech and language therapist visits to the service and the support provided the wider staff team.   **Team feedback:**   - Gather feedback from social care staff on the effectiveness and impact of speech and language therapist support in improving communication practices. - Method: questionnaires. - Explore experiences, challenges, and support needs around providing increased resource for communication. - Methods: In-depth interviews, focus group discussions. |
| **Speech and Language Therapists** have the capacity, knowledge and skills to implement improvements to communication support interventions. | B. Speech and language therapists are willing to support the development of communication skills and knowledge in social care services staff. | **[iii] Assessment of perceived knowledge and skills:**   - Gather insights from speech and language therapists to explore their self-assessed knowledge and skills in communication support. - Gather insights from direct support staff to explore their perceptions of speech and language therapists’ knowledge and skills in improving communication support within the staff team - Methods: Surveys, interviews, focus groups |
| **Speech and language therapists** are able to:   1) mentor staff in roles to increase resources for communication support 2) provide consultations with service managers 3) conduct assessments and develop interventions | H. Speech and language therapists are available and willing to provide mentoring and consultancy support. | **[ix] Competence measure:**   - Possible competency attributes: knowledge, skills, values, critical thinking, beliefs, judgement, attitude, ability, performance, research and evidence-based practice, leadership, mentoring and teaching, professional autonomy, quality management, promotion of/prioritisation of communication support. |
| **Social care services staff** are able to:   1) implement record keeping processes for communication support 2) share information on communication support efficiently 3) update communication resources efficiently | I. ICALD guidance for information and record sharing is compatible with existing data management systems of service organisations. | **[xi] Audit areas:**  **Quality of communication documentation:**   - Review verbal handovers, assessments, and communication documents for accuracy, relevance, and clarity for staff.   **Staff attendance at handover meetings:**   - Monitor and record attendance levels.   **Evidence of staff engagement with handover documents:**   - Use sign-in sheets or ‘read’ notifications to confirm documents have been reviewed. |
| **Adults with learning disabilities** are able to:   1) have more opportunities to express and have their needs met 2) have more opportunities to practice and improve their communication skills 3) receive timely improvements to their communication support |  | **[xiii] Observations**  Objectives:   - To assess the frequency of opportunities adults with learning disabilities have to express themselves, have their needs met, and opportunities to practice their communication skills with staff.   **Audit focus:**   - Review how often communication strategies are evaluated and adapted by staff. |
| **Long term goal**: Good communication between adults with learning disabilities and staff. |  | **[xiv] Observations:**   - Communication between adults with learning disabilities and staff. - Communication strategies and tools used by staff. - Level of engagement between adults with learning disabilities and staff.   **Performance review**  **Staff appraisals:**   - Include focus on communication support practices.   **Self- and peer evaluation:**   - Assess use of communication interventions. - Methods: Questionnaires, interviews. |
| **Impact**: Improved outcomes for adults with learning disabilities |  | **[xv] Audit**  **Identification and response to communication needs:**   - Whether the communication support needs of adults with learning disabilities are identified and appropriately addressed by support staff. - Method: Observations.     **Feedback from adults with learning disabilities:**   - Gather insights on satisfaction with communication support. - Methods: Interviews and supported conversations.   **Engagement with activities:**   - Levels of engagement and participation in activities by adults with learning disabilities. - Method: Observations. |
