# Supplementary material for: Developing a theory of change to improve the implementation of communication support for adults with severe learning disabilities in social care settings
Source: BMC Health Serv Res. 2026 Aug 6;26:1518. doi: 10.1186/s12913-026-15154-1 (PMC13733076; doi:10.1186/s12913-026-15154-1)
Supplement: Supplementary file 5 [file 12913_2026_15154_MOESM6_ESM.docx]

**Additional File 6: ToC components**

The impact of *improved outcomes for adults with learning disabilities* – which is beyond the ceiling of accountability (the number of outcomes that the implementation model can reasonably influence but cannot be solely responsible for achieving) – was agreed as the impact by participants. Discussions on deciding this were framed around the overall aim of the ICALD programme, which is to improve the implementation of communication support, and in turn, improve quality of life outcomes for adults with learning disabilities. This framing may have shaped how participants approached the discussion of the desired overall impact.

Participants agreed that the ultimate intended impact of the programme was the improved quality of life for adults with learning disabilities. However, they explicitly acknowledged that this goal sits beyond the ceiling of accountability (the maximum outcome the implementation model can reasonably influence but cannot be solely responsible for achieving).

### Long-term outcomes

Central to the long-term outcome of *good communication between adults with learning disabilities and staff,* is the development of effective, meaningful, and person-centred support that strengthens communication partnerships and addresses factors influencing the communication process. When social care staff are equipped with the appropriate communication skills, knowledge and strategies, participants believed adults with learning disabilities would be better able to participate in decision-making, meaningfully engage in social activities, have an improved understanding of daily routines supported by appropriate strategies, and have the means for expression.

### Medium- and short-term outcomes

Participants consistently emphasised that achieving meaningful improvements in communication support required early and widespread buy-in from all levels of social care staff. Leadership from senior organisational leads and service managers was seen as essential for driving change, as their visible commitment signified the importance of communication support as a strategic priority. Establishing this foundation in the *pre-implementation* phase was considered dependent on collaboration with key decision-makers and an informed understanding of available resources within each service context, including visual materials for communication and staff resources.

These actions were described as critical pre-conditions for ensuring that subsequent implementation efforts would be feasible and effective. Central to the implementation of the interventions are stakeholders including direct support staff, communication champions (or those in similar roles), service managers, organisational leads, SLTs, family carers, and adults with learning disabilities themselves. The professional expertise of SLTs and staff in role to provide additional communication resources were highlighted as a key resource for building capacity within social care teams. Participants noted that this required dedicated coaching and ongoing training opportunities to ensure individuals in Practice Leadership roles could effectively support staff through skills development and mentoring, thereby helping to minimise existing resource constraints on SLTs. Recruitment processes for direct support staff were also identified as areas requiring improvement, with suggestions that values-based hiring, competency assessments, and opportunities to shadow experienced staff would better ensure that new team members are equipped to prioritise high-quality communication support. Adults with learning disabilities and family carers were considered critical in co-development and co-facilitation of training, to ensuring that communication resources and training are person-centred.

Three pathways were identified as essential to achieving intermediate outcomes: (1) collaborative development of communication resources and training, (2) improved processes for information sharing and recording, and (3) ongoing skills development, peer mentoring and supervision. Participants stressed the need for changes to how communication strategies and tools, are developed and implemented, with particular attention to aligning resources with the communication preferences and needs of people with learning disabilities. Resource development coaching for family carers was proposed to enhance their involvement and ensure that strategies remain current and relevant. SLTs with specialist experience in improving communication support were seen as best positioned to lead these activities, with support from service managers and wider staff teams.

Linked to this, participants identified the need to revise current processes for updating communication resources and sharing information. Clearer guidance and coaching for staff on how to develop, implement, and share communication strategies were recommended to ensure they use accurate resources consistently and routinely share information within and between teams.

To improve the knowledge and skills of the workforce, participants advocated for the appointment of communication champions to act as Practice Leaders and provide coaching, peer support and modelling under SLT supervision. Reflective practice sessions, peer-learning, and supervision from SLTs and service managers, were regarded as essential mechanisms to promote a culture of ongoing learning and ensure that high standards of communication practice were embedded across all levels of service delivery.

Implementation strategies across the three pathways listed above, were seen to contribute to broader organisational outcomes, including systemic improvements in how changes to communication support are conceptualised, delivered, and sustained. Key changes involved stronger collaborative development of communication tools and training, including greater integration of expert input, and the establishment of clear procedures for sharing and recording communication needs. Participants felt that these developments would enable social care staff to embed best-practice communication approaches into daily interactions, thereby providing better support. Collectively these changes lead to indirect outcomes by strengthening communication partnerships, promoting conditions that enable adults with learning disabilities to have their needs acknowledged and appropriately supported.

### Assumptions

Participants identified several key conditions that they considered necessary for the intervention model to be successfully implemented. The research team used these insights to develop specific assumptions that described the circumstances needed for activities to lead to the intended outcomes. Participants highlighted the importance of both organisational and individual factors in reducing the risk of gaps in implementation. For example, they noted systemic issues such as limited supervision for social care staff? and limited access to up-to-date information and resources on communication support.

During the mapping process, each assumption was reviewed in terms of its likelihood, its potential influence on outcomes, and whether further action was required. In some cases, this meant considering additional interventions so outcomes across pathways could be achieved; in other instances, assumptions were judged to be reasonable without further intervention, or responsibility for implementing communication related interventions was allocated to stakeholders outside of the ICALD team (for example, organisations are responsible for monitoring staff communication practices).

The most significant assumptions identified by participants related to the availability of resources; the prioritisation of and commitment to enhancing communication support; the willingness and capacity of key stakeholders to engage in and sustain ICALD-related interventions; and the existing knowledge and skillsets of those responsible for implementing interventions. Challenges relating to commitment, consistency, and feasibility were identified as critical to achieving desired outcomes, particularly in under-resourced social care settings.

### Indicators and outcome measurements

Participants’ insights shaped the selection and refinement of indicators, ensuring that measurement approaches reflected priorities and challenges identified through their lived and professional experiences. Suggestions for measuring outcomes were discussed with participants, although no specific standardised, validated outcome measures were proposed.
